# Channeling and dampening: The role of political ties in information disclosure and concealment

**DOI:** 10.1371/journal.pone.0289016

**Published:** 2023-07-28

**Authors:** Weiting Zheng, Na Ni, Donal Crilly

**Affiliations:** 1 School of Management and Governance, UNSW Business School, UNSW, Sydney, Australia; 2 Faculty of Business, Lingnan University, Hong Kong, Hong Kong; 3 London Business School, London, United Kingdom; National Taiwan University, TAIWAN

## Abstract

Non-profit organizations (NPOs) help the state achieve its social objectives. At the same time, they often depend on the private-sector actors for donations. The different beliefs of public- and private-sector actors regarding which practices are desirable for NPOs can affect the transparency of these organizations. We propose that political ties influence NPOs to comply with state-mandated disclosure requirements, while simultaneously dampening their willingness to voluntarily disclose sensitive information that may jeopardize their legitimacy in the eyes of private-sector stakeholders. The impact of political ties on disclosure is contingent upon two factors. First, market institutions moderate such effects because expectations of public- and private-sector actors may diverge more in freer markets than where the state has inordinate power. Second, financial dependence on the state amplifies both effects as dependence on the state exerts more pressure for compliance whilst making politically connected organizations appear even more questionable in the eyes of the private-sector stakeholders. Leveraging a policy shock that weakened political ties, we found that following the policy shock, charities in China reduced their compliance to state-mandated information disclosure, but increased their voluntary disclosure. The opposing roles of political ties in mandatory versus voluntary disclosure is further supported by a policy capturing study involving private donors in China. This study has important implications for research on political ties and information disclosure.

*“The Oxfam scandal has taught us there is no reward for honest charities. NGOs are encouraged to skim over inconvenient facts and provide an immaculate account of success to win funding.”*

(The Guardian, January 22, 2013)

## Introduction

Non-profit organizations (NPOs) play a crucial role in helping governments achieve their social objectives [1]. At the same time, they must navigate relationships with private-sector actors, who are an important source of donations [2]. To win the confidence of supporters, NPOs must provide transparency [3, 4]. NPOs thus disclose information to signal their quality and good intent with the aim of reducing information asymmetry between them and external constituencies. This, in turn, may lead to greater legitimacy, support, and superior access to resources [5].

However, pursuing legitimacy may also be a reason to conceal information. Perceptions of legitimacy vary across actors [6, 7]. The state is evaluated for its own legitimacy, which denotes its accountability and its use and distribution of resources [8, 9]. But, the state also evaluates organizations as legitimate or otherwise [10], as do private-sector actors. Public- and private-sector actors may disagree about which behaviors are legitimate [11],. This gives rise to a dilemma when NPOs decide which kinds of information to disclose, especially when they have connections with the state through having government officials in the organization ([12, 13], hereafter, “political ties”). Political ties are generally perceived as legitimate by public-sector actors [14] but may signal poor governance to private-sector actors (i.e., donors), who worry that individuals connected with the state may use these connections for their own benefit [2]. Wanting to appear legitimate in front of both the public- and private-sector stakeholders potentially drives connected NPOs to disclose certain practices, but conceal others.

The question we address in this study is: *how do political ties influence organizations’ information disclosure*? We address this question by distinguishing different kinds of disclosure (mandatory vs. voluntary) and different sources of legitimacy—legitimacy in the eyes of the state and that in the eyes of private-sector. While mandatory disclosure refers to compliance with formal regulations [15], voluntary disclosure denotes conforming to “social and normative expectations not (yet) codified in standards and law” [16: 300]. Distinguishing different types of disclosure and sources of legitimacy challenges the dominant thinking that the search for legitimacy drives more disclosure. Instead, legitimacy concerns might discourage organizations from disclosing some information if it is viewed unfavorably by key audiences.

Our framework links political ties to *both* mandatory and voluntary information disclosure. We unearth two opposing roles of political ties. Political ties direct organizations to comply with mandatory disclosure (the “channeling” effect) while dissuading them from disclosing information outside of the state mandate (the “dampening” effect). Specifically, politically connected NPOs comply with the state’s mandate to maintain their political legitimacy; they bear higher costs of defiance due to their need to maintain political relationships and resource flows from the state. In contrast, disclosing non-mandated information may expose sensitive information (for example, the political affiliations of organizational leaders and financing sources) to donors, hurting their legitimacy in the eyes of the private-sector stakeholders. We also explore two important contingencies, namely how local contexts matter for audiences’ interpretation of information about an NPO’s political connectedness, as well as the relative importance of the public- and private-sector stakeholders to an NPO.

We apply this framework to examine the information disclosure of NPOs in China from 2010 to 2016. NPOs constitute an ideal context to test our theory as it is crucial for these organizations to navigate relationships with both the public- and private-sector actors (i.e., donors) [2, 17]. During our research period, an announcement issued by the Chinese Communist Party in June 2014 started restricting the ability of retired government officials to take up roles in social organizations. Leveraging this sudden announcement as an exogeneous policy shock to NPOs’ political ties, we found that Chinese charities reduced compliance with state-mandated information disclosure, but increased disclosing information outside the state mandate. These findings thus provide support for the opposing roles of political ties in mandatory versus voluntary disclosure. A supplementary policy capturing study ascertains the mechanisms that shape donor perceptions of politically connected organizations.

This study makes two core contributions. In delineating the channeling and dampening mechanisms of political ties on disclosure, we contribute to research on the interface between politics and organizations’ conduct [8, 18]. Whereas prior research reveals mixed relationships regarding political ties and organizational disclosure [19–21], we help resolve the contradictory findings by distinguishing state-mandated and non-mandated information disclosure, together with the respective approval that NPOs seek. Importantly, we show that political ties impede conformity to market expectations even though they drive compliance with state mandates. Relatedly, we contribute to disclosure research by examining the contexts influencing NPOs’ transparency. Going beyond existing literature that typically focuses on organization-level attributes [15], we highlight the institutional environment as an important boundary condition: evaluations of NPOs’ political ties are more negative in liberal environments, amplifying the opposing roles of political ties on disclosure.

## Political ties and information disclosure

Political ties are common across different political systems [13, 22, 23]. Prior research has produced mixed findings regarding the level of information disclosure by politically connected organizations. Government connections may motivate organizations to improve their disclosure [21], but some studies indicate that politically connected organizations are less transparent [19, 20].

Our starting point in reconciling these contradictory findings is the recognition that, although organizations often disclose information to pursue legitimacy, legitimacy “exists in the eye of the beholder” [24: 361]. Different audiences employ distinct criteria when deciding whether an organization is legitimate or not [6, 25]. Two actors are crucial for constructing NPOs’ legitimacy—the state and the donation market. NPOs rely on the state to regulate and approve their activity. They thus strive for legitimacy in the eyes of the state [26]. Simultaneously, they rely on private-sector transactional partners for financial resources and validation that they are using those resources appropriately [27]. The expectations of public- and private-sector actors regarding NPOs’ information disclosure may differ. The state is more concerned with ensuring that organizations comply with mandatory disclosure practices required by law, whereas private-sector stakeholders may demand overall transparency, including the state’s involvement in NPOs.

These divergent expectations create tension for politically connected NPOs. An NPO led by a public official may be concerned about maintaining approval from the state. It will be most motivated to disclose practices mandated by the state (Quadrant I, Table 1). Meanwhile, it may conceal information that the state has not mandated to disclose—especially where such disclosure might raise questions (Quadrant II, Table 1). Note that though *mandatory* implies an obligation to disclose information, in practice, organizations have discretion over compliance with formal regulatory requirements due to weak or incomplete enforcement [8]. On the other hand, an organization without ties to the state may be less motivated to disclose state-mandated practices. Its motivation is potentially tempered by concerns about revealing sensitive information to other NPOs in the same domain [28] (Quadrant III, Table 1). The unconnected NPO may still comply with mandatory disclosure requirements, but it is *comparatively* less likely to do so than a politically connected NPO, which has more to lose if it defies the state. Instead, compared with a politically connected organization, an unconnected organization may be more motivated to disclose those voluntary practices deemed necessary to secure legitimacy in the eyes of private-sector stakeholders (Quadrant IV, Table 1).

**Table 1:** Political ties and information disclosure.

	Mandatory disclosure	Voluntary disclosure
**Politically-connected organizations**	Quadrant I • Comparatively high • Mechanism of compliance, driven by need for maintaining legitimacy with the state • Higher costs of defiance due to their need to maintain resource flows from the state	Quadrant II • Comparatively low • Conformity is dampened by political ties, which might negatively impact legitimacy in the eyes of private-sector actors
**Non-politically-connected organizations**	Quadrant III • Comparatively low • Lower compliance as less pressure for securing political legitimacy (so the motivation to keep operational details secret from competition dominates)	Quadrant IV • Comparatively high • Mechanism of conformity, driven by need for legitimacy in the eyes of private-sector actors

Consequently, we argue that political ties in NPOs play a “channeling” role and induce compliance in disclosing *mandatory* information where the state has strong interests. In contrast, when it comes to *voluntary* information, political ties “dampen” the motivation of NPOs to disclose sensitive information that jeopardizes legitimacy in the eyes of the donors. Also, whether an organization pays greater attention to the state or market varies according to context. Strong market institutions reduce the channeling effect of political ties, but amplify the dampening effect due to more divergent interests between the public- and private-sector actors and, thus, more negative evaluations of NPOs’ political connectedness.

We develop our theory around NPOs in China because such organizations are susceptible to the divergent pressures mentioned above. The government mandates NPOs to declare specific information in their audited annual reports by following a government-approved template (similar to the IRS Form 990 for non-profits in the U.S.) through specified channels. Non-compliant NPOs face the risk of pecuniary sanctions or of being prohibited. Still, disclosure on the mandated items falls short of where it should be due to weak legal enforcement. At the same time, market forces (wherein donors are a key actor) have been at work to shape the disclosure landscape. As a result of the growth in philanthropy, private donors increasingly request information from NPOs to inform their donation decisions.

## Hypotheses

### Political ties and information disclosure

Governments seek to improve the transparency and accountability of NPOs [29], be it to achieve a better-functioning non-profit sector or stronger state control. The government establishes policies to advance NPOs’ transparency on various dimensions. Mandating disclosure represents an extension of the government’s political legacy in the non-profit sector [30]. Governments look favorably on compliant NPOs as they help achieve the state’s goals. NPOs, in turn, face the expectations of the state to demonstrate regulatory compliance.

Politically-connected NPOs face stronger pressure to comply with the state mandate. Due to the influence of their political careers and networks, leaders of politically connected NPOs are acutely aware of the benefits of complying with government regulation and the cost of defiance. Consequently, through their presence in the leadership teams of NPOs, politicians have the opportunities and power to channel the state’s expectations and demand regulatory compliance. In addition, politically-connected NPOs bear higher costs of defiance due to the possibility of losing their political legitimacy and the resource flow from the state. Whereas financing is essential for NPOs to fulfill their missions, the state also wields substantial influence in the domain of non-financial resources—especially in its control of the registration process that NPOs must abide by as well as its influence over NPOs’ perceived legitimacy [31]. Politically connected organizations depend disproportionally on the state for permits and subsidies [32]. Simply put, connected NPOs have more at stake if they fail to comply. For instance, ties to public officials subject for-profit firms in China to greater political scrutiny in adopting state-initiated CSR disclosure [33], and such ties in Taiwan allowed government officials to obtain greater compliance from connected firms in adopting contested corporate governance practices [34]. In contrast, organizations without political ties, whilst still incentivized to pursue political legitimacy, have less to lose and thus a lower risk from not fully complying [33].

Taken together, we conclude that the motivation to maintain political legitimacy drives greater compliance with state mandates for politically tied organizations. Hence, we propose,

**Hypothesis 1 [channeling effect].** Political ties are positively associated with organizations’ compliance with mandatory information disclosure practices.

Whilst Hypothesis 1 is consistent with the prior literature, we go further to argue that political ties can even disincentivize NPOs to disclose information that is not mandated by the state. With the privileged access to resources provided by political ties, connected organizations are less sensitive to market norms than those without political ties [19]. In addition, we propose that political ties can even make voluntary disclosure *riskier* because such disclosure might expose sensitive information. Political linkages can lead to favors being exchanged between political actors and the focal organization [35]. Connected insiders may advance personal interests by bestowing favors on politicians in return for benefits [36], installing unqualified allies in the organization [37], or pursuing private gains [38]. Disclosing related information may expose organizations to greater public scrutiny. Politically connected organizations thus have less incentive to disclose information voluntarily to the public.

As an example, it is not a state-mandated requirement for NPOs in China to report the political affiliations (if any) of their board members, though many civil society actors want such information to be made public. The General Secretary from a large politically connected NPOs we interviewed commented,

*“(Politically-connected) NPOs may not disclose much of their finance management rules, as it may clearly show that how they are heavily influenced by the governmental supervision unit…The same goes for the C.V. of the General Secretary, which may also expose the person’s political linkage and lack of professional experience…”*

In sum, political ties dampen the motivations of organizations to disclose information voluntarily. We thus propose that,

**Hypothesis 2 [dampening effect].** Political ties are negatively associated with organizations’ conformity with voluntary information disclosure practices.

### The moderating role of market institutions

In explaining heterogeneity in information disclosure, the existing literature tends to focus on organization-level attributes [15]. Whereas we consider (below) a critical organizational moderator, i.e., the extent to which an organization depends on the state for resources, institutional settings are also likely to moderate the influence of political ties on information disclosure by shaping the perceptions of the public- and private-sector actors.

Where the state enjoys inordinate authority over market activities, market functioning is subject to greater political influence so that expectations of the public- and private-sector actors tend to converge. In contrast, expectations diverge more in freer markets governed by market principles. In markets such as the U.S., government control of business is typically considered illegitimate [39] while state-owned enterprises loom large in other economies such as China. Importantly, however, within China subnational governments have substantial discretion over economic policy, and there is substantial variation in how markets function [40].

For mandatory disclosure, if NPOs disclose information to secure political legitimacy, the channeling effect of political ties is likely weaker in freer markets. Where market institutions function well, they can serve as an alternative channel to drive information disclosure. These pressures—both regulatory and normative pressures—directly drive compliance with regulations [41], reducing the need for the channeling role of political ties. As such, even politically unconnected organizations are likely to comply. In contrast, in contexts where government authority over the market is significant, market pressures are likely to be too weak to drive compliance. Political ties, in such markets, thus play a more active role in driving compliance. Hence, in such contexts, the channeling effect of political ties is amplified. Therefore, we posit:

**Hypothesis 3a.** The positive relationship between political ties and organizations’ compliance with mandatory information disclosure practices is weaker in markets that are relatively independent of state influence.

For voluntary disclosure, as the dampening effect of political ties arises from the different expectations of public- and private-sector actors, this effect is stronger where divergence of expectations is greater, i.e., in contexts with freer market institutions. Although private-sector actors demand transparency and we might therefore expect politically connected organizations to behave similarly to their unconnected peers in free-market contexts, private-sector actors are also more skeptical of political ties. Links between business and political leaders are commonly questioned in free markets due to perceived conflicts of interests and the potential for abuse of power [42]. Similarly, donors are more averse to the political connections of NPOs because such connections are viewed as incompatible with market principles. As a result, the heightened liability of political connectedness further disincentivizes politically connected organizations from voluntarily disclosing information. Such information is often sensitive in nature, such as when organizations are required to name their most important funding sources and leaders’ political affiliations.

In contrast, political ties are more prevalent–and may be viewed as normatively appropriate—in contexts where the state enjoys disproportionate influence over market activities [12, 13], which softens the negative perception of political ties. Here, market donors are also more aware of the benefits that political ties offer [2], countering the negative perception they may form of political ties. This is reflected from our interviews of donors in China, as evidenced in the following quote,

*“(Political ties) may make the organization lose its uniqueness (independence), … But they will also enhance the credibility of the organization and allow it to better integrate with the government’s initiatives than grassroot charities.”*

Therefore, we propose that

**Hypothesis 3b.** The negative relationship between political ties and organizations’ conformity with voluntary information disclosure practices is stronger in markets that are relatively independent of state influence.

### The moderating role of organizations’ financial dependence on the state

An organization’s information disclosure not only depends on the legitimacy judgments of its stakeholders, but also on how consequential their judgments are. We posit that the positive effect of political ties on compliance is stronger for organizations with more revenue sourced from the state, such as government subsidies. Under such circumstances, securing legitimacy and related benefits from the state is more important, and the cost of non-compliance is also greater.

Meanwhile, if political ties dampen the motivation to voluntarily disclose information because being transparent about political connectedness risks legitimacy in front of donors, the dampening effect will be stronger for organizations that depend on the state for financial resources. Here, the risk of connected insiders seeking private benefits (or, the perception of such risks) is arguably higher [38, 43], and the disincentive to disclose information voluntarily is thus larger for the connected organizations. Therefore, connected NPOs that depend heavily on the government may be even more reluctant to disclose information voluntarily—especially, where such disclosure relates to sensitive topics.

Thus, we expect that financial dependence on the government will strengthen the positive (negative) relationship between political ties and mandatory (voluntary) information disclosure.

**Hypothesis 4a.** The positive relationship between political ties and organizations’ compliance with mandatory information disclosure practices is stronger for organizations that depend more on the state for financial resources.

**Hypothesis 4b.** The negative relationship between political ties and organizations’ conformity with voluntary information disclosure practices is stronger for organizations that depend more on the state for financial resources.

## Research design

### Data and sample

Our empirical study is based on a unique proprietary database of Chinese philanthropic foundations from 2010 to 2016. The year 2010 is the first year for disclosure data to be made available. Disclosure data are provided by the China Foundation Center (CFC), the pioneering organization that is committed to the higher transparency of philanthropic foundations in China (more information about the CFC’s data collection is available in [44]. As the most influential independent rating agency of philanthropic foundations in China, the CFC collects extensive disclosure data using foundations’ publications, government civil affairs agencies at the national and local levels, and specific media. The percentage of foundations covered by the CFC out of the overall population exceeds 85 percent. The audited annual reports provide information for all other foundation-level variables. The analysis period provides a longitudinal setting to examine information disclosure for foundations in China. The initial sample encompasses an unbalanced panel of 2,709 foundations between 2010 and 2016 (inclusive).

### Measures

#### Dependent variables

In measuring information disclosure, we used the Foundation Transparency Index (FTI), which tracks a comprehensive set of disclosure items by Chinese foundations and has been annually updated by the CFC since 2010. The FTI identifies 41 disclosure items, including both mandated and voluntary items. Details of the FTI are described in Appendix A in S1 File. These 41 items are largely consistent with the items used in prior investigations of Chinese foundations (e.g., [44]).

*Mandatory disclosure*. An item was counted as mandatory disclosure if it was required by the Ministry of Civil Affairs to appear in a foundation’s annual report. Table 2 provides a list of disclosure items. If a mandated item was disclosed, it was coded as “1”, and “0” otherwise. *Mandatory disclosure* was measured annually by the ratio of the summed value of these disclosed items for a foundation (which ranges from 0 to 33) to the total number of items mandated by the state in that year. Higher ratios indicate higher levels of mandatory disclosure. Due to the relatively weak implementation of laws and regulations, despite the mandatory nature of these practices, on average, about 80% of these items were disclosed. Fewer than 20% foundations had full compliance. Replacing the ratio measure of disclosure with count measure does not change our results (results available from authors).

**Table 2:** Mandatory and voluntary disclosure items in Chinese philanthropic foundations, 2010–2016.

Mandatory items	Voluntary items
1. mission	1. project management rule ^a^
2. original endowment fund value	2. major donor ^b^
3. full time employee no.	3. finance management rule ^b^
4. telephone no.	4. cv of general secretary
5. board member names	5. original endowment funder
6. address	6. board member affiliation
7. donation income	7. existence of a charter (i.e., a formal document stating the operational scope and management rules)
8. charitable expenditure	
9. total assets	8. project showcase on the website
10. net assets	9. donor query page on the website
11. total income	10. existence of website
12. investment returns	11. whether there is dedicated information release page on website
13. government fund	
14. service delivery income	
15. total expenses	
16. salary expenses	
17. admin expenses	
18. operating costs	
19. management expenses	
20. fundraising expenses	
21. project expenses	
22. project income	
23. project name	
24. project description	
25. fund usage	
26. project venue	
27. operation domains	
28. annual report	
29. personnel management rule	
30. project management rule ^a^	
31. major donor ^b^	
32. finance management rule ^b^	
33. audit report	

^a^ A voluntary disclosure item during 2010 and was mandated since 2011; coded as voluntary disclosure for 2010 and mandatory disclosure for 2011 and the following years

^b^ A voluntary disclosure item during 2010–2011 and was mandated since 2012; coded as voluntary disclosure for 2010–2011 and mandatory disclosure for 2012 and the following years.

*Voluntary disclosure*. Voluntary disclosure items were those that were not mandated in annual reports but were among the FTI items. If such an item was disclosed by a foundation, it was coded as “1”, and otherwise “0”. *Voluntary disclosure* was then measured by the ratio of the summed value of these disclosed items for a foundation (which ranges from 0 to 11) to the total number of voluntary disclosure items in that year. Higher ratios indicate higher levels of voluntary disclosure by foundations. On average, less than half of the voluntary items were disclosed. Replacing the ratio measure of disclosure with count measure does not change our results.

#### Independent variable

Following previous research [14, 45], we recorded a *political tie* if a charity had government officials(s) serving in the senior leadership team. We collected the curricula vitae of each foundation’s senior leaders (i.e., Chairman, Vice-chairman, and General-secretary) from the website of Baidu Baike (http://baike.baidu.com/), a large data source that provides the curricula vitae of Chinese business and political leaders. We complemented this source with foundations’ websites, annual reports, audit reports, and other major information platforms (such as CSMAR). In total, we compiled 14,676 distinct names for foundation leaders. We collected the political ties data for each foundation annually as senior leaders may change over time. We scrutinized each person’s C.V. to determine whether they had or currently served as a government official. We focus on political ties to governmental agencies because, compared to legislative bodies in China, the government controls critical resources (e.g. regulation, budgets, licenses and permits) and create substantial resources dependence for organizations [46]. We recorded a foundation’s political ties as a count variable based on the number of ties identified using the above-mentioned process. We identified forty three percent (43%) of cases in the initial sample as politically connected, with the number of political ties ranging from 0 to 18. As robustness checks, we tested the existence of political ties as a dummy variable.

#### Moderator

We adopted the marketization index for Chinese provinces to measure market institutions. This index is updated annually by the National Economic Research Institute [47], and has been frequently used in studying China’s institutional development [48]. The index reports differences in institutional development across China’s 31 provinces and municipalities. To capture market independence from government intervention, we used the sub-index that evaluates market institutions related to state-market relations (which includes measures on “percentage of resources allocated by the market”, “constraints on government intervention in business”, and “government scale”). As such, a higher value on *market independence* indicates greater independence from the government in a province. In our robustness analyses, we replaced the sub-index of *market independence* with the overall marketization index (correlation = 0.78).

*State subsidies* was calculated as the percentage of total state subsidies received to the total revenues of the charity. About 22% of foundations received state subsidies. We also adopted a dichotomous measure, based on whether the foundation received state subsidies in a given year, in our robustness analyses.

#### Control variables

We included a set of control variables that might influence NPOs’ information disclosure. We measured a foundation’s *size* using its total assets (in the logarithm form) annually [32]. *Age* was measured by the number of years since its establishment [29]. *Donation ratio* controlled for financial dependence on the donation market, and was calculated as the total sum of private (i.e., non-state) donations received divided by the total revenue of a foundation, annually. As a control for other political affiliation, *government founder* was coded “1” if the foundation’s founder involved a major political or quasi-political entity (for details, see [32]); otherwise, it was coded “0”. Consistent with existing research [49], we distinguished having a government founder from personal-level political ties because the latter may reflect a different dimension of political embeddedness [50]. *Public qualification* is a dummy variable that took the value of “1” if a foundation had the right to conduct public fundraising and “0” if it could only raise funds privately.

*Audit quality* measures the governance quality of the foundation and reflects whether the auditor for a foundation is in the annual ranking of China’s “top 100” reputed accounting firms. The ranking is published by the Chinese Institute of Certified Public Accountants [51]. The name information is retrieved from the audited annual report of foundations; the dummy variable *audit quality* takes the value “1” if a foundation’s auditor appears in the ranking list, and “0” if it does not. Furthermore, we included *service domain dummies* to reflect the primary service domain. The operations of Chinese philanthropic foundations are categorized into seven distinct domains: (1) the arts, culture, and humanities, (2) education, (3) environment and animals, (4) health, (5) human services, (6) foreign affairs, and (7) public and societal benefits. To control for prior performance in overall disclosure, we included the *FTI score* received by a foundation from the CFC. The FTI score was normalized for ease for comparison across years. To control for the potential inter-related nature of *mandatory* and *voluntary disclosure*, we controlled for the lagged mandatory disclosure in models predicting voluntary disclosure, and vice versa.

We also controlled for macro-environmental factors that influence disclosure. *Density of foundations* controls for the competitive pressure on foundations in a region and was calculated as the number of foundations divided by the total population in a province (or a direct-administered municipality) in China. Finally, we included *year* fixed effects to control for trends.

### A quasi-natural experiment analytical approach

We leveraged a policy change in our research context, testing its influence on the impact of political ties. It had been common for government officials to take part-time positions in Chinese NPOs. This practice was widespread until June 2014 when the Chinese Communist Party issued *The Notice of Regulating the Re-employment of Retired Officials to Part-time Positions in Social Groups*. This policy began to restrict retired officials from taking positions in non-profits. Not anticipated by the public, this new policy created an exogenous shock to NPOs’ existing political ties. On the one hand, the channeling effects of political ties on mandatory disclosure will be weakened as a result of the change. On the other hand, restrictions on the active involvement of political officials may have reduced negative perceptions of political ties, weakening the dampening role of political ties on voluntary disclosure. As a result, by testing how the policy shock influenced the impact of political ties, we are able to gauge the actual impact of political ties on foundation disclosure.

Admittedly, the presence of political ties was not randomly assigned among Chinese philanthropic foundations. To address this concern, we draw upon prior studies (e.g., [52]) in using propensity score matching (PSM) to construct a control group with characteristics similar to those of our treatment group (i.e., foundations with political ties) [53]. This approach requires calculating each foundation’s propensity score for receiving the treatment (i.e., being politically connected). We used logistic regression and all the control variables to predict each foundation’s propensity score. Next, we matched each politically connected foundation with a control that was not politically connected but has a very similar propensity score to the treatment foundation. We used 1:1 nearest-neighbor matching (without replacement) with a caliper of 0.05 [54, 55]. As expected, after matching, the differences between the connected and unconnected foundations become non-significant in all dimensions except for political ties (see Appendix B in S1 File). Unmatched cases were dropped, resulting in a test sample of 1,768 observations.

Next, we ran panel data fixed-effects regressions on the matched sample. All independent and control variables are lagged by one year in the analyses. Our model specification is as follows:

$${Disclosure}_{i,t}=\beta_{0}+\beta_{1}{Political\;ties}_{i,t-1}+\beta_{2}{Post2014}_{i,t}+\beta_{3}{Political\;ties}_{i,t-1}\times {Post2014}_{i,t}+{Controls}_{i,t-1}+\mu_{t}+{ƹ}_{i,t},$$ (1)

where *Disclosure*_*i*,*t*_ is the dependent variable of mandatory or voluntary disclosure for a foundation in a given year. *Political ties*_*i*,*t*−1_ is the treatment group; *Post*2014_*i*,*t*_ equals 1 if a year is after the policy change, i.e., years 2015 and 2016. The interaction term, *Political ties*_*i*,*t*−1_×*Post*2014_*i*,*t*_, is the variable of interest: a negative coefficient, *β*_3_, for the equation of mandatory disclosure would indicate that the effect of political ties was weakened following the policy shock, supporting our prediction that political ties have a positive effect on mandatory disclosure, whereas a positive coefficient of *β*_3_ for the equation of voluntary disclosure would suggest a strengthened effect of political ties following the policy shock, supporting our prediction that political ties have a negative impact on voluntary disclosure. *Controls*_*i*,*t*−1_ include all control variables described above, *μ_t_* denotes year fixed-effects, and ƹ_*i*,*t*_ is the error term. Given that our key variable of interest is an interaction term, for ease of interpretation, we test the moderating effects by conducting split-sample analyses. Details of the subsample constructions are reported in the following Results section.

## Results

Table 3 depicts the descriptive statistics of our matched sample and the correlations between the variables that feature in our models. On average, for our matched sample, 85.7% of the mandatory items have been disclosed, whereas 41.9% of the voluntary items were disclosed. 51.6% of the foundations have political ties, and the number of political ties ranges from 0 to 13, with an average value of 0.743. The independent variables are only moderately correlated.

**Table 3:** Summary statistics and correlations.

Variables	(1)	(2)	(3)	(4)	(5)	(6)	(7)	(8)	(9)	(10)	(11)	(12)	(13)	(14)
1 Mandatory disclosure	1.000													
2 Voluntary disclosure	0.206	1.000												
3 Political ties	-0.005	0.096	1.000											
4 Market independence	-0.041	0.033	-0.073	1.000										
5 State subsidies	-0.072	-0.025	0.016	0.009	1.000									
6 Government founder	0.054	0.006	0.013	-0.026	0.011	1.000								
7 Audit quality	0.120	0.226	0.162	0.004	-0.030	0.046	1.000							
8 Size	0.178	0.277	0.044	0.097	0.032	0.152	0.199	1.000						
9 Age	0.079	0.045	0.014	0.029	0.017	0.171	0.045	0.117	1.000					
10 Public status	0.059	-0.003	-0.005	-0.088	0.131	0.406	0.011	0.107	0.276	1.000				
11 Donation ratio	-0.025	0.251	-0.028	0.039	0.062	-0.040	0.023	0.115	-0.046	0.010	1.000			
12 Foundation density	0.103	0.441	0.077	0.176	0.038	-0.092	0.312	0.254	0.020	-0.113	0.202	1.000		
13 Region disclosure	0.168	0.126	0.055	0.044	-0.067	0.071	0.253	0.158	0.089	0.061	-0.059	0.100	1.000	
14 FTI score	0.325	0.605	0.030	0.013	-0.021	-0.004	0.214	0.317	0.063	-0.009	0.174	0.335	0.150	1.000
Mean	0.857	0.419	0.743	4.199	0.044	0.801	0.146	16.875	11.693	0.671	0.855	0.049	48.15	57.77
S.D.	0.179	0.311	1.083	2.639	0.156	0.399	0.354	1.813	11.88	0.47	1.045	0.042	5.604	19.494
Min.	0.031	0	0	-4.8	0	0	0	0	1	0	0	0	13.317	4.8
Max.	1	1	13	13.23	1	1	1	22.118	32	1	1.0	0.17	59.813	100

N = 1,768 (matched sample)

Table 4 presents the results of the fixed-effects regressions testing Eq 1. The dependent variable in models 1–5 is the percentage of mandatory disclosure practices that the focal foundation implemented; in models 6–10, it is the percentage of voluntary disclosure practices. Models 1 and 6 test the main effect of political ties respectively, models 2–5 and 7–10 use split-sample analysis to test the moderating effects. Hypotheses 1 and 2 predict a positive effect of political ties on mandatory disclosure and a negative effect of political ties on voluntary disclosure, respectively. Following our quasi-natural experiment approach, we expect the interaction term, political ties * post2014, to have a negative effect on mandatory disclosure, and a positive effect on voluntary disclosure. The results in columns 1 and 6 support both predictions. Political ties* post2014 has a negative effect on mandatory disclosure (column 1: β = -0.01, s.e. = 0.007, *p* = 0.058), and a positive effect on voluntary disclosure (column 6: β = 0.02, s.e. = 0.009, *p* = 0.010). In terms of economic significance, one additional political tie in a foundation increases the percentage of mandatory disclosure by 0.01, which is 5.6% (0.01/0.179) of 1 standard deviation of mandatory disclosure (= 0.179). And, one additional political tie in a foundation reduces the percentage of voluntary disclosure by 0.02, which is 6.4% (0.02/0.311) of 1 standard deviation of voluntary disclosure (= 0.311). Hypotheses 1 and 2 are supported.

**Table 4:** Fixed-Effects models estimating political ties and information disclosure of Chinese philanthropic foundations, 2010–2016.

	DV: mandatory disclosure					DV: voluntary disclosure				
	(1)	(2)	(3)	(4)	(5)	(6)	(7)	(8)	(9)	(10)
	H1	H3a market independence		H4a state subsidies		H2	H3b market independence		H4b state subsidies	
		High	Low	High	Low		High	Low	High	Low
**Political ties** *	**-0.01+**	**-0.003**	**-0.03** *	**-0.08+**	**-0.01**	**0.02** *	**0.04+**	**0.03**	**0.06+**	**0.03** **
**post2014**	(0.058)	(0.636)	(0.032)	(0.086)	(0.166)	(0.010)	(0.053)	(0.183)	(0.065)	(0.006)
Post2014	0.87***	-0.03	-0.30***	0.67*	0.88***	-1.41***	-0.02	0.20***	-2.74***	-1.38***
	(0.000)	(0.154)	(0.000)	(0.011)	(0.000)	(0.000)	(0.596)	(0.000)	(0.000)	(0.000)
Political ties	-0.00	-0.00	-0.00	-0.16**	-0.00	0.00	-0.01	0.01	-0.02	0.01
	(0.727)	(0.498)	(0.981)	(0.004)	(0.972)	(0.525)	(0.329)	(0.552)	(0.480)	(0.262)
Market independence	0.00			0.00	-0.00	-0.00			0.03**	-0.00
	(0.576)			(0.736)	(0.677)	(0.510)			(0.007)	(0.640)
State subsidies	0.00	0.01	0.05			0.01	0.02	-0.00		
	(0.939)	(0.632)	(0.115)			(0.801)	(0.847)	(0.978)		
Government founder	-0.01	-0.04	-0.07+	0.08	-0.01	0.01	0.04	0.02	-0.13	0.01
	(0.730)	(0.289)	(0.078)	(0.287)	(0.825)	(0.551)	(0.321)	(0.630)	(0.172)	(0.498)
Audit quality	-0.03	0.02	-0.17*	-0.07	-0.06	-0.06+	-0.02	-0.06	-0.21	-0.07+
	(0.467)	(0.372)	(0.038)	(0.359)	(0.171)	(0.068)	(0.597)	(0.351)	(0.110)	(0.051)
Size	-0.00	-0.00	-0.01	0.01	-0.00*	0.01*	0.00*	0.01	0.01	0.01*
	(0.164)	(0.529)	(0.126)	(0.389)	(0.040)	(0.034)	(0.011)	(0.116)	(0.851)	(0.032)
Age	-0.21***	0.02*	0.01	-0.11+	-0.21***	0.34***	-0.01	0.02+	0.61***	0.33***
	(0.000)	(0.012)	(0.241)	(0.090)	(0.000)	(0.000)	(0.308)	(0.061)	(0.000)	(0.000)
Public status	0.03	-0.04	0.08		0.05+	0.03	0.07	-0.16	0.75***	-0.01
	(0.450)	(0.402)	(0.629)		(0.085)	(0.726)	(0.201)	(0.461)	(0.000)	(0.848)
Donation ratio	0.02***	-0.00	0.02***	-0.03	0.03***	-0.04***	-0.01	-0.03**	-0.09***	-0.03**
	(0.000)	(0.661)	(0.000)	(0.274)	(0.000)	(0.000)	(0.109)	(0.003)	(0.000)	(0.001)
Foundations density	0.07***	0.01	0.06*	0.03	0.09***	0.00	0.05	-0.00	0.05	0.00
	(0.000)	(0.247)	(0.016)	(0.720)	(0.000)	(0.763)	(0.291)	(0.833)	(0.508)	(0.841)
FTI score	-0.00*	-0.00	-0.00*	-0.00	-0.00**	0.00	0.00*	0.00	0.00	0.00
	(0.024)	(0.989)	(0.014)	(0.407)	(0.009)	(0.526)	(0.019)	(0.252)	(0.693)	(0.740)
Voluntary disclosure	0.08	-0.01	0.25**	-0.26	0.15*					
	(0.304)	(0.851)	(0.002)	(0.267)	(0.010)					
Mandatory disclosure						-0.34**	-0.22+	-0.44+	0.19	-0.36**
						(0.002)	(0.090)	(0.060)	(0.372)	(0.002)
Year dummies	Yes	Yes	Yes	Yes	Yes	Yes	Yes	Yes	Yes	Yes
Constant	3.18***	0.83***	1.71***	1.97*	2.88***	-2.66***	0.06	0.96***	-7.43***	-2.47***
	(0.000)	(0.000)	(0.000)	(0.013)	(0.000)	(0.000)	(0.756)	(0.000)	(0.000)	(0.000)
Observations	1,768	763	1,005	181	1,587	1,728	769	959	181	1,547
R-squared	0.24	0.24	0.32	0.46	0.24	0.45	0.13	0.50	0.74	0.45

Robust p-value in parentheses

*** p<0.001

** p<0.01

* p<0.05

+ p<0.1

Hypotheses 3a and 3b examine the contextual contingency of Hypothesis 1 and 2, and predict that market independence from state interference will reduce the channeling effect while strengthening the dampening effect of political ties. To test this set of hypotheses, we split the sample into sub-samples of high vs. low market independence based on the mean value of *market independence*. Results in columns 2–3 show that, for mandatory disclosure, Political ties * post2014 has a significantly negative effect for the sub-sample of low market independence (column 3: β = -0.03, s.e. = 0.015, *p* = 0.032), whereas such effect is not present for the sub-sample of high market independence (column 2: β = -0.003, s.e. = 0.007, *p* = 0.636). The contrast of effects supports H3a that the channeling effect of political ties on mandatory disclosure is stronger in contexts with lower market independence. On the other hand, results in columns 7–8 show that, for voluntary disclosure, political ties * post2014 has a positive (albeit marginally significant) effect for the sub-sample of high market independence (column 7: β = 0.04, s.e. = 0.019, *p* = 0.053), whereas no significant effect has been found for the sub-sample of low market independence (column 8: β = 0.03, s.e. = 0.020, *p* = 0.183). The contrast of the effects supports H3b that the dampening effect of political ties on voluntary disclosure is more salient in markets with higher independence from state interference.

Hypotheses 4 examine the moderating effects of government subsidies to assess whether NPOs that depend on the state for financing will exhibit greater compliance to state mandate and reluctance in voluntary disclosure. Again, we split the sample into two sub-samples of high vs. low dependence on state subsidies based on the mean value of *state subsidies*. For mandatory disclosure, results in columns 4–5 show that political ties * post2014 has a negative effect for foundations with higher state subsidies (column 4: β = -0.08, s.e. = 0.046, *p* = 0.086), while political ties * post2014 has no effect for foundations with lower state subsidies (column 5: β = -0.01, s.e. = 0.010, *p* = 0.166). These results support H4a that the channeling effect of political ties on mandatory disclosure is stronger when the organizations have greater dependence on financial subsidies from the state. Turning to voluntary disclosure, results in columns 9 and 10 show that political ties * post2014 has a positive effect for both sub-samples with higher and lower levels of state subsidies. H4b is not supported, indicating that politically connected NPOs tend to engage in less voluntary disclosure regardless of their financial dependence on the state.

It is worth noting that foundations established by the government do not disclose more state-mandated information. This suggests that the state founder represents a type of political affiliation that is distinct from the affiliation that individual-based political ties give rise to, confirming the value of investigating political ties separately. In addition, interestingly, foundations that disclose more mandatory items disclose fewer voluntary items. These results confirm that mandatory and voluntary disclosure are different in nature, and organizations respond to these disclosure practices differently.

### Mechanism test: A policy capturing study to validate mechanism of H2

An assumption underlying our theory is that private donors may negatively evaluate informational cues pertaining to organizations’ political connections and level of professionalization (which are voluntary dimensions of disclosure). To support this argument, we conduct a policy capturing study. Policy capturing is apt to identify how informational cues shape audiences’ assessments of organizations [56]. Subjects evaluate fictive organizations that are constructed to allow researchers to manipulate variables of interest.

Our aim was to select a sample that was fairly representative of China-based donors. With that in mind, we partnered with Credamo, a China-based company with access to screened research participants, to recruit 204 professionals to participate in an online study. All had previously donated to philanthropic organizations. We screened for prior work experience. 124 (60.78%) participants had worked exclusively in the private-sector, and 80 participants were working, or had worked, in the state sector. 83 (40.69%) participants declared membership of the Chinese Communist Party (CCP), two did not declare their membership status, and the remainder were non-members. In our analysis, we include the two participants who did not declare membership as non-members. Excluding these two participants does not materially change our results. 97 (47.55%) participants were female. 185 (90.68%) were university graduates. Participants were distributed across 24 provinces and municipalities. The two most represented provinces, Guangdong and Jiangxi, differ substantially in the strength of local market institutions.

We created descriptions of philanthropic foundations that were identical except for four dimensions: *domain*, *audit report*, *donor query*, and *General Secretary C*.*V*. The *domain*, i.e., service domain of the foundation, was described as raising China’s global profile in the medical domain (0) or community regeneration (1). These are consistent with two of the seven domains for foundations operating in China and reflect the declared purposes of foundations in our archival data. The *audit report*, a mandatory reporting requirement for foundations, was listed as unavailable (0) or available (1). The *donor query facility*, consistent with transparency and voluntary disclosure, was listed as non-existent (0) or existent (1). *General Secretary C*.*V*., the core cue of interest and a key voluntary disclosure item, was described as unavailable, an official with professional (i.e., philanthropic sector) experience, or an official with a political background. All descriptions and instruments were initially created in English and subsequently translated into Chinese.

Because our study has three variables—*domain*, *audit report*, *donor query*—with two values each, and a fourth variable—*General Secretary*—which has three values, we have 24 permutations in total. Showing subjects all 24 permutations can cause fatigue [57] (Mitchell, Shepherd, Sharfman 2011). We thus presented each subject with six randomly selected scenarios from the 24. For each scenario, subjects scored their *willingness* to donate on a scale from 1 (very low) to 7 (very high). To capture various dimensions of perceived legitimacy, we also asked them to indicate on the same scale (1) the degree to which they thought the foundation would use donated funds wisely, (2) the degree to which it was trustworthy, and (3) the degree to which the organization’s principles matched those of the respondent. Each of these items reflects a somewhat distinct dimension of legitimacy. Organizations perceived as legitimate offer appropriate accounts of what they do [58], are viewed as trustworthy [7], and audiences perceive shared understandings about basic principles of operating [59].

We report the details of our analyses and results in Appendix C in S1 File. In a nutshell, foundations that disclose the professional background of their General Secretary to private donors are at an advantage to those that disclose a political background. Specifically, donors have lower confidence in such charities’ wise use of funds and trustworthiness, look askance at their principles, and are less likely to donate to them.

### Robustness analyses

We conduct several additional analyses that aim to ensure the robustness of our primary results. To explore the possibility that information disclosure level may influence foundations’ establishment of political ties, we used the change of political ties between periods *t* and *t*+1 as the dependent variable and disclosure (both mandatory and voluntary) in period *t* as the independent variable. Neither mandatory nor voluntary information disclosure significantly affects the formation of political ties (Model 1, Appendix D in S1 File), suggesting that reverse causality is not a concern.

In addition, we adopted alternative measures and methods to validate the robustness of our results. First, we combined mandatory and voluntary items and created an overall disclosure variable for each foundation. The effects of political ties on this new dependent variable were no longer discernible, highlighting the value of examining mandatory and voluntary disclosure practices separately (Model 2, Appendix D in S1 File). Second, we tested the political tie variable as a dummy variable; results are consistent with those reported (Models 3 and 4, Appendix D in S1 File). Third, some mandated items involve core identifying information that foundations readily make public and have little to do with disclosure, such as address and telephone number. We excluded these “easy to disclose” items related to basic facts and focused on items related to financial, governance, and program information, i.e., items with “high disclosure content”. We excluded five items in this analysis: mission, original endowment fund, telephone number, address, and board member names. All five items have a disclosure rate higher than 95%. Results were qualitatively the same as those reported in Table 4 (not tabulated). Finally, we performed itemized tests for all voluntary disclosure practices, and found a negative effect for almost all items (exceptions include charter and major donor).

## Discussions and conclusions

This study is motivated by mixed predictions about the influence of political ties on organizations’ information disclosure. We bring together two factors that have not received adequate attention. First, the common wisdom is that the pursuit of legitimacy drives information disclosure [60]. Yet, as public- and private-sector actors frequently disagree about which practices matter, pursuing legitimacy can lead politically-affiliated NPOs to conceal certain information. Second, not all forms of information disclosure are equally important to all organizations. The state has an interest in their compliance with its mandate, which compels politically-connected NPOs to comply with mandated disclosure practices (“channeling effect”). In contrast, political ties may be viewed negatively by private-sector stakeholders, discouraging NPOs from conforming with voluntary disclosure standards that involve potentially sensitive information (“dampening effect”).

Our findings provide strong support that Chinese NPOs’ political ties have opposing effects depending on whether disclosure is mandatory or voluntary in nature. Evidence from the policy capturing study suggests that the opposing effects of political ties on mandatory versus voluntary information disclosure are consistent with different audiences’ perceptions of legitimacy. Our tests of boundary conditions also confirm that, where market institutions are more independent of state interference and, thus, expectations of the public- and private-sector actors diverge, the channeling effect of political ties on mandatory disclosure is weaker, whist the dampening effect is stronger.

This study contributes to scholarship on the relationship between political ties and organizational transparency. Existing research has provided mixed findings about this relationship. While some studies suggest that political ties may motivate organizations to improve their disclosure [21], others indicate that politically connected organizations are less transparent [19, 20]. Extending this literature, our study provides evidence as to how political ties can simultaneously channel and dampen information disclosure in organizations, depending on whether the disclosure is mandated by the state or voluntary. This distinction between state-mandated and voluntary disclosure reflects divergent expectations from different stakeholders (public- versus private-sector actors), a perspective largely absent in the literature (see also [2]). The study thus highlights the need for political strategy research to consider the interest of the state and private-sector actors and how they may diverge. Whereas the state seeks to ensure compliance with mandatory disclosure standards, private-sector stakeholders desire other forms of transparency that can even undermine the interests of the state—such as when private-sector stakeholders demand that NPOs declare their political affiliations.

For mandatory disclosure, where pressure from the state is strong, the need to maintain political legitimacy created by political ties restricts discretion and induces organizational compliance. For voluntary disclosure, political ties potentially convey unfavorable information to private-sector actors, leading politically connected organizations to conceal information. These findings resolve previous mixed results on the influence of political ties on information disclosure, extending the focus of research from examining whether “political ties buffer organizations from or bind organizations to the government” [48] to considering the perceptions of a broader set of stakeholders beyond the state. Our conclusion is that political ties induce organizations to comply with pressures emanating from the state and hinder them from conforming with pressures emanating from the market.

Relatedly, our findings have implications for government policies promoting organizational disclosure. Pursuing political legitimacy may sometimes encourage concealment of certain practices, highlighting the double-edged role of privileged relations with the government. This approach paints a fuller picture of the impact of political ties on governance practices, by simultaneously considering mandatory and voluntary governance practices and the distinct pressures that underlie them. Policy makers should be aware of this when deciding which practices to make mandatory. It is taken for granted that making practices mandatory fosters compliance [15]. Yet, governments should be aware of the Janus role of their linkages to organizations: while such linkages can induce organizations to pursue politically favored goals, they may simultaneously hinder organizational adoption of market-demanded practices that are conducive to a higher governance standard.

Our study offers important directions for future research. Future research can extend our framework to other contexts (for example, outside China and considering organizations other than NPOs). For example, for-profit companies also have relations with the state, and it would be productive to investigate how they balance pressures for compliance with the state mandate and pressures for conformity to market expectations. To investigate such questions, scholars could implement a policy capturing approach similar to the method we used in our second study. Future studies in distinct contexts might extend, and investigate the boundary conditions to, our theory.

## Supporting information

S1 File (PDF)

S2 File (PDF)

S1 Data (DTA)
