# Supplementary material for: Channeling and dampening: The role of political ties in information disclosure and concealment
Source: PLoS One. 2023 Jul 28;18(7):e0289016. doi: 10.1371/journal.pone.0289016 (PMC10381070; doi:10.1371/journal.pone.0289016)
Supplement: S1 File — (PDF) [file pone.0289016.s001.pdf]

Supporting Information

S1. APPENDIX A. The Composition of the Foundation Transparency Index (FTI)

The CFC provides the most comprehensive information on philanthropic foundations in China, including the composition of the FTI since 2010. To meet growing needs from multiple stakeholders (e.g., government, individual and institutional donors, media, universities and research institutes), FTI ranks all Chinese foundations against a comprehensive checklist of transparency items over years in the four categories of general information, financial information, project information, and donor information. To improve the index, the CFC invited over 70 consultants that are professors from major universities and senior leaders from NPOs, and iterated the revision process in several rounds of work. The FTI ranking is updated annually based on the database maintained by the CFC.

Details regarding FTI are described as follows:

	Year 2010	Year 2011	Year 2012	Year 2013	Year 2014
Number of total foundations tracked by FTI annually	1,832	2,214	2,580	3,043	3,582
Annual population of foundations	2,156	2,548	2,994	3,580	4,216
Percentage of foundations covered by FTI	84.97%	86.89%	86.17%	85.00%	84.96%
Full score of all four categories in FTI	129.4	129.4	107.2	100	100

The computation of FTI total score follows this formula: $\mathbf{FTI_n = \sum(T_i \times W_i \times S_i \times C_i)}$

- FTI_n: the transparency score of foundation n;
- n: the identifier of foundations;
- i: the identifier of disclosure items;
- T_i: Equals 1 if the foundation discloses item i, otherwise 0;
- W_i: Weight, the weight of item i in calculating the total score, which is between 1 and 6. Higher value means more weight;
- S_i: Source, the channel of information disclosed, for which 1.2 represents foundation website, and 0.8 for other channels;
- C_i: Coverage, the degree of completeness of item i, which ranges from 0 to 1. Higher value means higher coverage. (Note: This component is only relevant for items concerning project information)

S2. APPENDIX B. Effectiveness of Propensity Score Matching

Variables	Unmatched / Matched	Mean: Treated	Mean: Control	%reduction |bias|	t-test: t	t-test: p>t
Size	U	16.985	15.826		16.83	0.000
	M	16.973	16.969	99.7	0.04	0.965
Age	U	11.944	7.7672		14.42	0.000
	M	11.907	12.024	97.2	-0.23	0.820
Public status	U	.68421	.40379		17.92	0.000
	M	.68332	.6918	97	-0.42	0.674
Donation ratio	U	.89646	.936		-1.04	0.301
	M	.89636	.94158	-14.3	-0.89	0.375
Service domain (1) the arts, culture, and humanities	U	.15414	.09595		6.04	0.000
	M	.15457	.13949	74.1	0.98	0.327
Service domain (2) education	U	.42199	.56698		-9.14	0.000
	M	.42319	.41942	97.4	0.18	0.860
Service domain (3) environment and animals	U	.05357	.03905		2.31	0.021
	M	.05372	.04713	54.6	0.69	0.488
Service domain (4) health	U	.21241	.17216		3.30	0.001
	M	.21301	.22432	71.9	-0.63	0.529
Service domain (5) human services	U	.31955	.23879		5.86	0.000
	M	.31762	.35156	58	-1.66	0.098
Service domain (6) foreign affairs	U	.06861	.01362		12.91	0.000
	M	.06598	.06409	96.6	0.18	0.860
Density of foundations	U		-0.22691		4.50	0.000
	M		-0.15239	88.5	0.33	0.741
Average region disclosure	U	48.265	47.536		4.35	0.000
	M	48.253	47.899	51.4	1.48	0.139
FTI score	U	58.881	51.991		11.45	0.000
	M	58.787	58.45	95.1	0.39	0.699

S3. APPENDIX C. Policy Capturing Study to Validate Mechanism of H2

Our analysis progresses in three stages. First, we regress the willingness to donate to the focal foundation on the scenario cues. We include rater fixed effects as each subject evaluates six foundations. In this initial analysis, we code whether the foundation discloses the C.V. of the General Secretary. We present our results in Table C1. All disclosure cues (except for *domain)* load positively on the propensity to donate and are associated with more positive perceptions of the foundation in question.

Table C1. Results of Policy Capturing: All Disclosure Cues and Donors' Propensity to Donate

	(1)<br>Propensity to donate
Domain	-0.075
	(0.257)
Donor query	1.019
	(0.000)
Audit report	0.996
	(0.000)
General Secretary C.V.	0.567
	(0.000)
Constant	3.000
	(0.000)
N	1224
Wald chi2(4)	660.77

P-values in parentheses

Crucially, disclosing the C.V. of the General Secretary, however, implies one of two things: disclosing a General Secretary with a political background, or disclosing a General Secretary with a relevant professional background. Hence, in the second stage, we repeat the analysis for the foundations disclosing this information but include an indicator for whether the General Secretary has a political background (1) versus a professional background (0). This is our central comparison because of our interest in comparing the perceptions of foundations with political ties vs. those without. Moreover, in our study, subjects are explicitly told whether an item of information is available or not. On average, they are likely to welcome more disclosure because an organizational explicitly refusing to disclose information is presumed to have something to hide. In the field, an organization does not make explicit that it refuses to disclose. We present our results in Column 1 of Table C2. Here, the coefficient on the General Secretary having a political background is negative and significant for the propensity to donate ($\beta = -0.209$, $p = 0.010$). In short, foundations that disclose the professional background of their General Secretary to private donors are at an advantage to those that disclose a political background.

Notably, this analysis contains donors that may be skeptical of the state as well as those who are affiliated with the state in some way. Thus, we break our sample into two groups: those who are members of the CCP and those who are non-members. As is clear from the results in Columns 2 and 3 of Table C2, it is non-members who react somewhat negatively to a General Secretary with a political background ($\beta = -0.188$, $p = 0.077$), whereas CCP members are less likely to distinguish between a General Secretary with a political background and a General Secretary with a professional background ($\beta = -0.177$, $p = 0.142$).

Table C2. Results of Policy Capturing: General Secretary's Background and Donors' Propensity to Donate

	(1)<br>Propensity to donate	(2)<br>Propensity to donate<br>(CCP non-members)	(3)<br>Propensity to donate<br>(CCP members)
Domain	-0.090	-0.281	0.095
	(0.266)	(0.008)	(0.429)
Donor query	0.883	0.978	0.713
	(0.000)	(0.000)	(0.000)
Audit report	1.026	1.250	0.663
	(0.000)	(0.000)	(0.000)
Political vs. professional background of General Secretary	-0.209	-0.188	-0.177
	(0.010)	(0.077)	(0.142)
Constant	3.856	3.725	4.212
	(0.000)	(0.000)	(0.000)
N	807	471	327
Wald chi2(4)	290.51	251.55	61.81

P-values in parentheses

Finally, in the third stage, we conduct mediation analyses to assess whether perceptions of donation use, trustworthiness, and shared principles mediate the effect of disclosing a General Secretary with a political background versus a General Secretary with a professional background on the propensity to donate. As in the previous stage, we conduct this analysis separately for CCP-members and CCP-non-members. To do so, we use Generalized Structural Equation Modeling (GSEM) in Stata which allows for multiple observations per respondent. Consistent with Preacher and Hayes (2008), we use bootstrapping with 500 replications. This permits us to assess the indirect effects of perceived donation use and shared principles without assuming that these effects are normally distributed (MacKinnon, Lockwood, and Williams, 2004). Our mediation model is shown in Figure C1. In calculating each path, we also control for all other information cues (i.e., the domain of the foundation; whether there is an audit report; whether there is a donor query function).

Figure C1. Policy Capturing Mediation Model

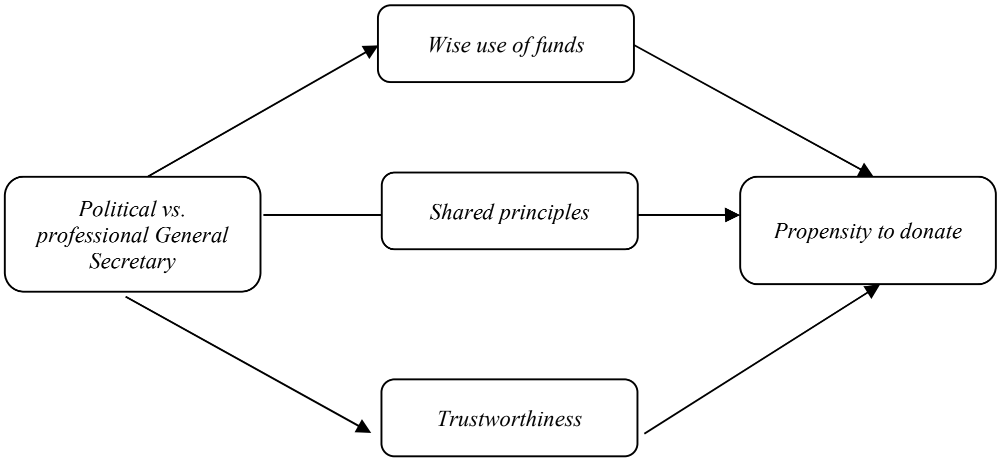

For the sub-sample of CCP members, consistent with our analysis above there is no direct effect on willingness to donate of disclosing an official with a political connection versus an official with a professional background. Moreover, there is no evidence of an indirect effect via perceptions of superior use of funds ($\beta = 0.036$, $p = 0.269$), shared principles with the donor ($\beta = 0.014$, $p = 0.474$), or trustworthiness ($\beta = 0.028$, $p = 0.558$).

For the sub-sample of CCP non-members, once perceptions of fund use, shared principles and trustworthiness are included in predicting the propensity to donate, the direct effect of disclosing an official with a political connection versus an official with a professional background finds no support ($\beta = 0.037$, $p = 0.585$), implying full mediation. We find support for the indirect pathway through the perceived wise use of funds ($\beta = 0.098$, $p = 0.013$), with the bootstrap estimate of the 95% confidence interval ranging from 0.021 to 0.177. We find marginal support for the indirect pathway through perceived shared principles ($\beta = 0.040$, $p = 0.082$), with the bootstrap estimate of the 95% confidence interval ranging from -0.005 to 0.0848. We find no support for the indirect pathway through perceptions of trustworthiness ($\beta = 0.060$, $p = 0.212$), with the bootstrap estimate of the 95% confidence interval ranging from -0.035 to 0.155.

S4. APPENDIX D. Robustness Analyses

	A. Test Reverse Causality	B. Combine mandatory and voluntary disclosure	C. Measure political ties as a dichotomous variable	
Dependent variable	Change in political ties	Total disclosure	Mandatory disclosure	Voluntary disclosure
	(1)	(2)	(3)	(4)
Political ties * post2014		0.01	-0.01*	0.02**
		(0.474)	(0.032)	(0.007)
Post2014		-0.59***	0.86***	-1.41***
		(0.001)	(0.000)	(0.000)
Political ties		-0.00	0.01	-0.01
		(0.932)	(0.443)	(0.676)
Mandatory disclosure	-0.77			
	(0.152)			
Voluntary disclosure	-0.10			
	(0.779)			
All control variables	Included	Included	Included	Included
Observations	1,768	1,768	1,768	1,728
R-squared	0.04	0.15	0.24	0.45

p-value in parentheses; *** p<0.001, ** p<0.01, * p<0.05, + p<0.1
