# Supplementary material for: Channeling and dampening: The role of political ties in information disclosure and concealment
Source: PLoS One. 2023 Jul 28;18(7):e0289016. doi: 10.1371/journal.pone.0289016 (PMC10381070; doi:10.1371/journal.pone.0289016)
Supplement: S2 File — (PDF) [file pone.0289016.s002.pdf]

Description of the Project

Subjects will be recruited via email and the survey will be administered entirely online. Therefore, the following information page will be the first page of the online survey, and informed consent will be obtained by the participant clicking on “yes” at the bottom of the page and continuing with the survey.

You are invited to take part in a research study being conducted by Na Ni (Shenzhen University) Weiting Zheng (UNWS Business School), and Donal Crilly (London Business School). The information presented on this page is provided to help you decide whether or not to take part in this study. If you decide to take part in this study, you will indicate that you give your consent to participate by clicking on the “Yes” button at the bottom of this page. If you decide you do not want to participate, there will be no penalty to you.

The purpose of the study is to learn more about the factors that influence a person’s perception of information disclosure by charities in China. You are being asked to participate in this study because you currently reside in China. To participate, all you will be asked to do is to complete an online survey which we anticipate will take approximately 20 minutes or less to complete. The bulk of the questions in this survey will be separated into assessing some factors within two broad areas. The first area is related to some of the information about disclosure practices of simulated charities in your community and how they influence your evaluation; and the second area is related to your own demographics as an individual.

Aside from your time, there are no other costs for taking part in this study, and the risks of participating are no more than what you would encounter in everyday life. Also, you will not be compensated for participating in this study. Once you have completed the survey, all identifying information including your name and email address will be discarded and you will not be asked to do anything further as part of this study. We assure you that all of the information contained in the survey and the performance assessment are strictly confidential.

People who have access to the survey responses include the researchers for this project. They access your responses to make sure the study is being run correctly and that information is collected properly.

Again, your participation is voluntary and you may withdraw from the study at any time. If you have any questions about the study, the Co-Investigator, Na Ni would be happy to talk with you. You can email her at na.ni@szu.edu.cn. For questions about your rights as a research participant; or if you have questions, complaints, or concerns about the research, you may contact the Institutional Review Board at Shenzhen Audencia Business School of Shenzhen University at (755) 2653 2363 or wangtianyu@szu.edu.cn.
